# Ptychographic analysis of human bone marrow‐derived mesenchymal stem cell morphology: The impact of cell senescence

**DOI:** 10.1111/jmi.70003

**Published:** 2025-07-11

**Authors:** Lorenzo Anconelli, Giovanna Farruggia, Isabella Zafferri, Francesca Borsetti, Stefano Iotti, Francesca Rossi, Jeanette A. Maier

**Affiliations:** ^1^ Department of Pharmacy and Biotechnology University of Bologna Bologna Italy; ^2^ INBB – Biostructures and Biosystems National Institute Roma Italy; ^3^ Dipartimento di Scienze Biomediche e Cliniche Università di Milano Milan Italy; ^4^ Centre for Applied Biomedical Research – CRBA Bologna Italy

**Keywords:** dry mass, mesenchymal stem cells, morphology, motility, ptychography, senescence

## Abstract

Mesenchymal stem cells (MSC) undergo replicative senescence, a state of irreversible cell cycle arrest that limits their utility in regenerative medicine applications. To identify novel markers of senescence useful to enhance the quality of MSC‐based therapies, we compared young and senescent human bone marrow‐derived mesenchymal stem cells (hMSCs) using a non‐invasive, label‐free approach based on quantitative phase imaging (QPI) with the Livecyte microscope. Senescent hMSCs demonstrated substantial morphological alterations, including a threefold increase in cell area, elevated dry mass, reduced thickness, and decreased sphericity compared to their younger counterparts. Additionally, motility metrics such as instantaneous velocity and displacement were significantly reduced in senescent cells, underscoring functional impairments that could hinder their therapeutic potential in regenerative medicine. The application of QPI offers a promising tool for monitoring cellular health, identifying, and potentially eliminating, senescent cells to improve the quality and effectiveness of MSC‐based therapies.

## 1 INTRODUCTION

Cells imaging is of fundamental importance in biological research. Since its invention in the 18th century, microscopy has been an indispensable tool in both medicine and biology. Over its long history, this technique has undergone continuous evolution, enabling increasingly detailed insights into cellular structures and processes. Today microscopy is still evolving, driving new discoveries and advancing research in diverse biological fields.

Quantitative phase imaging (QPI) is one of the emerging techniques in cell research that enables the measurement of fundamental cell properties and behaviours, including mass, volume, morphology, growth and intracellular transport.^1^, ^2^ QPI is based on measuring the phase shift of light as it passes through a transparent sample. This phase shift occurs due to the slowing down of light in a material with a higher refractive index than water.^3^ The measured phase shift is directly proportional to the dry mass content of the biological sample and this ability to evaluate quantitative biophysical features of the cells is fundamental to its application in cellular biology.

Classical methods, such as phase‐contrast microscopy and differential interference contrast (DIC), are designed to enhance the visibility of transparent or weakly absorbing samples by converting phase shifts into intensity differences. They are widely used in biological research to observe live cells without staining, providing information about cell morphology, motility, and other properties. However, these techniques typically rely on classical light sources and do not involve quantum properties of light, providing a qualitative assessment of the image. On the contrary, QPI allows for highly accurate quantification of cellular properties like mass, volume, and intracellular transport.^1^, ^2^

Furthermore, QPI offers several advantages over conventional imaging approaches. It does not require fixation, extensive sample preparation, or exogenous labelling agents, enabling the monitoring of subtle changes in live cells over extended periods, by leveraging the diffraction of light. Unlike ‘classical’ phase contrast or interference microscopy, QPI does not require special lenses to detect small differences in nearly transparent structures, such as cells. Additionally, the use of low‐intensity light sources minimises photodamage, making QPI particularly advantageous for live‐cell imaging.^4^ The main problem that QPI techniques have to address is how to quantify the information contained in the phase shift. In this context, several approaches have been proposed, such as the use of interferometry, wavefront sensing, phase retrieval, digital holography (for an exhaustive review see Nguyen 2022) and ptychography.^5^

Ptychography can reconstruct the complex wavefront of the light emerging from the sample. It separately retrieves the amplitude and phase of both the sample and the probe, providing high‐quality quantitative phase information.^6^, ^7^ It uses multiple diffraction patterns collected from spatially overlapping regions of the sample to form images using iterative algorithms.^8^ These algorithms perform a refinement of the amplitude and phase estimate by utilising the diffracted intensities and knowledge of the real‐space position of each overlapping point in the scan. For this reason the retrieval of the phase information from ptychography is of very high quality, and visible‐light ptychography permits to obtain label‐free imaging of cells,^5^, ^9^ allowing, for example, to discriminate between healthy cells and cancer cells,^10^ and to detect morphological changes in cells treated with anticancer drugs.^11^, ^12^

The morphologies of live cells undergo significant alterations in response to disease states such as viral infections, cancer, or to gradual deterioration of functional characteristics as in senescence.^4^ For instance, measurements of cell thickness using QPI have revealed a characteristic phenotype in cancer cells^13^, ^14^ and documented variations in the morphology of human A549 alveolar epithelial cells infected with H3N2 influenza viruses.^4^ Senescence, in particular, induces volume changes and irregular morphologies in several cell types,^15^, ^16^ which may be driven by cytoskeletal modifications.^17^, ^18^ The state is also associated with an increased abundance of RNA and proteins, potentially linked to altered RNA turnover and protein degradation.^19^ Despite these insights, the phenotypes associated with cellular senescence are highly variable and heterogeneous, although they exhibit certain common features.^16^ However, the mechanisms underlying these shared markers remain incompletely understood.

The study of aging in human mesenchymal stem cells (hMSCs) is of fundamental importance since these cells are widely used in tissue engineering and cell therapy.

The need to generate large quantities of hMSCs necessitates their maintenance in culture for multiple passages over extended periods. However, prolonged culture can lead to the accumulation of senescent cells, which may contribute to the development of significant pathological conditions.^20^ It has been suggested that selective ablation of senescent cells could enhance the therapeutic efficacy of hMSCs.^21^, ^22^ Thus, identifying morphological hallmarks of aging in these cells is essential for therapeutical applications.

In this study, the Livecyte microscope (Phase Focus, Sheffield, UK) was employed. This microscope utilises ptychography to evaluate cells, generating high‐contrast, label‐free images, enabling detailed analysis of cellular morphology and behaviour.^5^

## 2 MATERIAL AND METHODS

### 2.1 Cell culture

hMSCs isolated from the bone marrow of healthy female donors were obtained from Clinisciences (Amsterdam, Netherlands), and kept in culture in DMEM medium (GIBCO, Life Technology Limited, Paisley, UK) added with 20% Fetal Bovine Serum (FBS) (GIBCO, Life Technology Limited, Paisley, UK), Penicillin 100 U/mL (Merck KGaA, Darmstadt, Germany) and Streptomycin 0.10 mg/mL (Merck KGaA, Darmstadt, Germany). hMSCs were rendered senescent by serial passages in vitro.^15^, ^17^

### 2.2 QPI analysis

The Livecyte Kinetic Cytometer (Phasefocus, Sheffield, UK) was employed to observe cell morphology and motility over a 20‐h period, with images captured at 30 min intervals using a 10× magnification, with the software Acquire v 3.10.6. The time interval limited to 20 h has been chosen to limit any interference in the evaluated parameters of any mitotic cells. However, considering that Young (Y) cells have duplication times that are around 48 h and that Senescent (S) cells present a proliferative block, virtually no mitotic cells were detectable in our sample.

To perform the QPI analysis, the cells were detached and seeded in a black μ‐Plate 24 wells with high quality plastic bottom designed for confocal microscopy or QPI microscopy (Ibidi Gmbh, Lochhamer Schlag, Grafelfing, Germany, Cat.No:82426). After 24 h the plate was placed in the Livecyte microscope equipped with a CO_2_ incubator maintained at 37°C and 5% CO_2_ and 4 frames per well were chosen. The frames surrounded a 1 mm^2^ surface each and were placed trying to choose well area in which the cells were well separated. The analysis was performed by Livecyte Phasefocus’ Cell Analysis Toolbox software v 3.12.2 and by GraphPad Prism 10 software.

The parameters chosen for these analyses were Area, Dry Mass, Volume, Thickness, Sphericity, Instantaneous Velocity, Track Speed and Displacement. The detailed description of the calculation of the parameters is reported in Supplementary Information S1.

The data are reported as the mean value calculated for each cell during the 20‐h period at each interval (40 data points). The cells were firstly automatically segmented and then a manual refinement was performed. The segmentation mask is reported in Supplementary Information S2.

### 2.3 Statistical analysis

Statistical analysis was performed by unpaired *t*‐test with the Mann–Wh**i**tn**e**y *U*‐test (Wilcoxon rank‐sum) correction tests (*p* < 0.05), considering the not‐normal data distribution. Three samples for both cell conditions were examined. Data, reported in a table as median ± IQD (interquartile difference), could be found in Supplementary Material S3.

## 3 RESULTS

### 3.1 Cell morphology

It is well established that hMSCs undergo morphological changes when they become senescent after serial passages in vitro.^23^ For this study, we utilised the Livecyte microscope, which provides information by analysing the diffraction of light passing through the sample.

**FIGURE 1:**
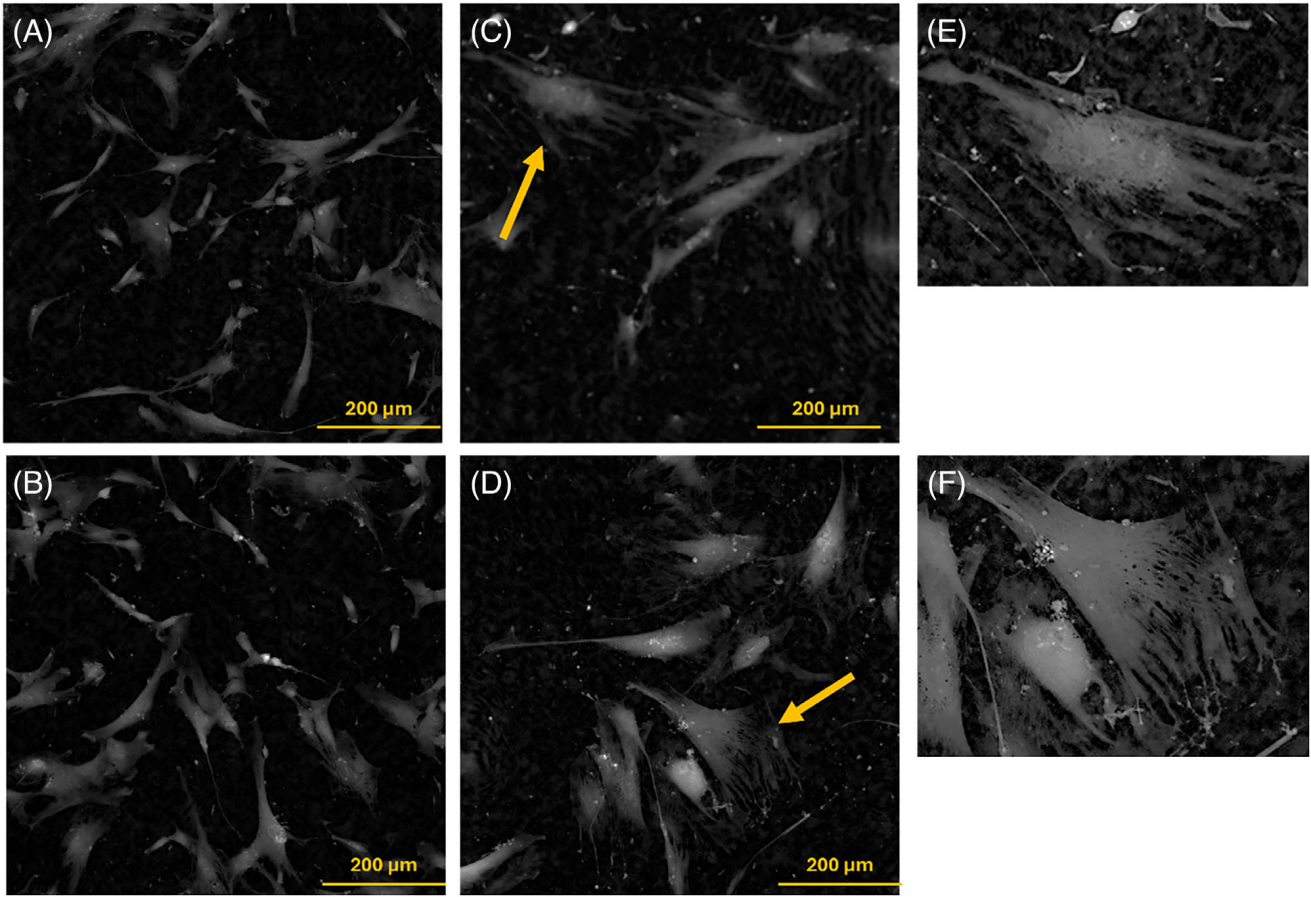
Ptychographic Images of Y (A and B) and S (C and D) hMSCs. In E and F, the two times enlargement of the two S cells indicated with the yellow arrows in C and D are reported.

Figure 1 shows images of Young (Y) (Figure 1A and B) and Senescent (S) (Figure 1C and D) cells. Notably, S cells appear larger than the Y counterpart, and characterised by irregular morphology with numerous extensions, as evidenced in Figure 1E and F.

To gain deeper insights into these morphological changes, we take advantage of the fact that QPI technique enables the acquisition of quantitative data on numerous cellular characteristics, such as cell size, morphology and motility in living cells, without perturbation.^1^, ^4^, ^5^

Cell area, measured as the cumulative number of pixel included within a feature's segmentation boundary and multiplied by the pixel size of the image, is statistically different between Y and S cells. Specifically, the median cell area of Y cells is approximately 1217 µm^2^, whereas S hMSCs have an median area of about 3544 µm^2^, making S cells more than three times larger than their young counterparts. Furthermore, the Area plot indicates also that S cells present a wider distribution, indicating a noticeable heterogeneity in morphology.

**FIGURE 2:**
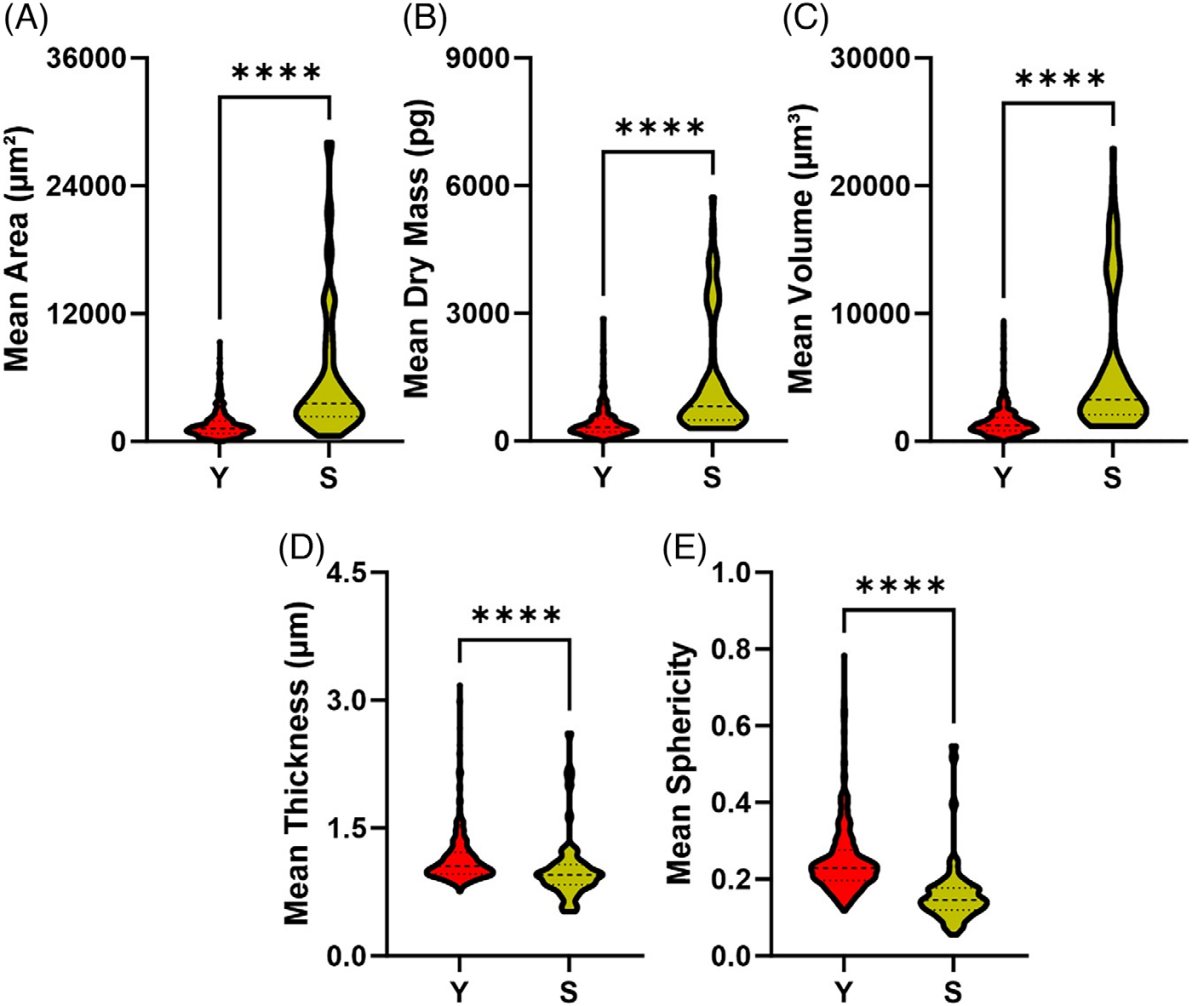
Violin plots representing the distributions of cell area (A), dry mass (B), volume (C), thickness (D) and sphericity (E). These plots highlight the differences in morphology between Y and S cells. The dashed lines within the violin plots indicate the median values of the distributions, while the dotted lines represent the quartiles. Significant differences between S and Y cells are indicated as ****p < 0.05.

Notably, the dry mass, which is a measure of the total non‐water cellular matter, reported in Figure 2B, increases 3.4‐fold in S compared to Y cells, with a median value of 813 pg versus 322 pg in Y hMSCs. These results are in agreement with the literature, where an increase in intracellular material is reported.^15^, ^16^ This direct measurement of dry mass is only possible with label‐free QPI technology, a result that supports the notion that S cells remain metabolically active, although they don't proliferate. These results are consistent with those obtained by Liu X et al., who reported that senescence induced in MDCK cells with the genotoxic drug doxorubicin for 5 days leads to a dramatic increase in dry mass.^24^ Additionally, similar findings are reported by Bresci A et al.,^25^ who observed increases in dry mass and flatness in desferoxamine‐induced senescence of Hep G2 cells.

Since volume and dry mass are calculated based on the measured PhaseValue (see Supplementary S1), they can be considered related, as dry mass represents the scaled version of volume. In the Livecyte analysis, the volume distribution in S and Y cells exhibits similar trends to dry mass one (Figure 2B). However, S cells are thinner, with a median thickness of 0.95 µm versus 1.05 µm of Y cells (Figure 2C), and they are less spherical, as indicated by a lower sphericity value (0.150 vs. 0.229) (Figure 2D).

### 3.2 Cell motility

Motility is a critical feature of hMSCs, as cell migration plays a vital role in maintaining bone health. For instance, abnormal MSC migration has been implicated in bone diseases such as osteoporosis.^26^ Moreover, enhancing cell migration has been proposed as a potential therapeutic strategy for treating bone disorders.^26^, ^27^ Senescence is known to reduce hMSCs motility, as reported by He et al.^28^

**FIGURE 3:**
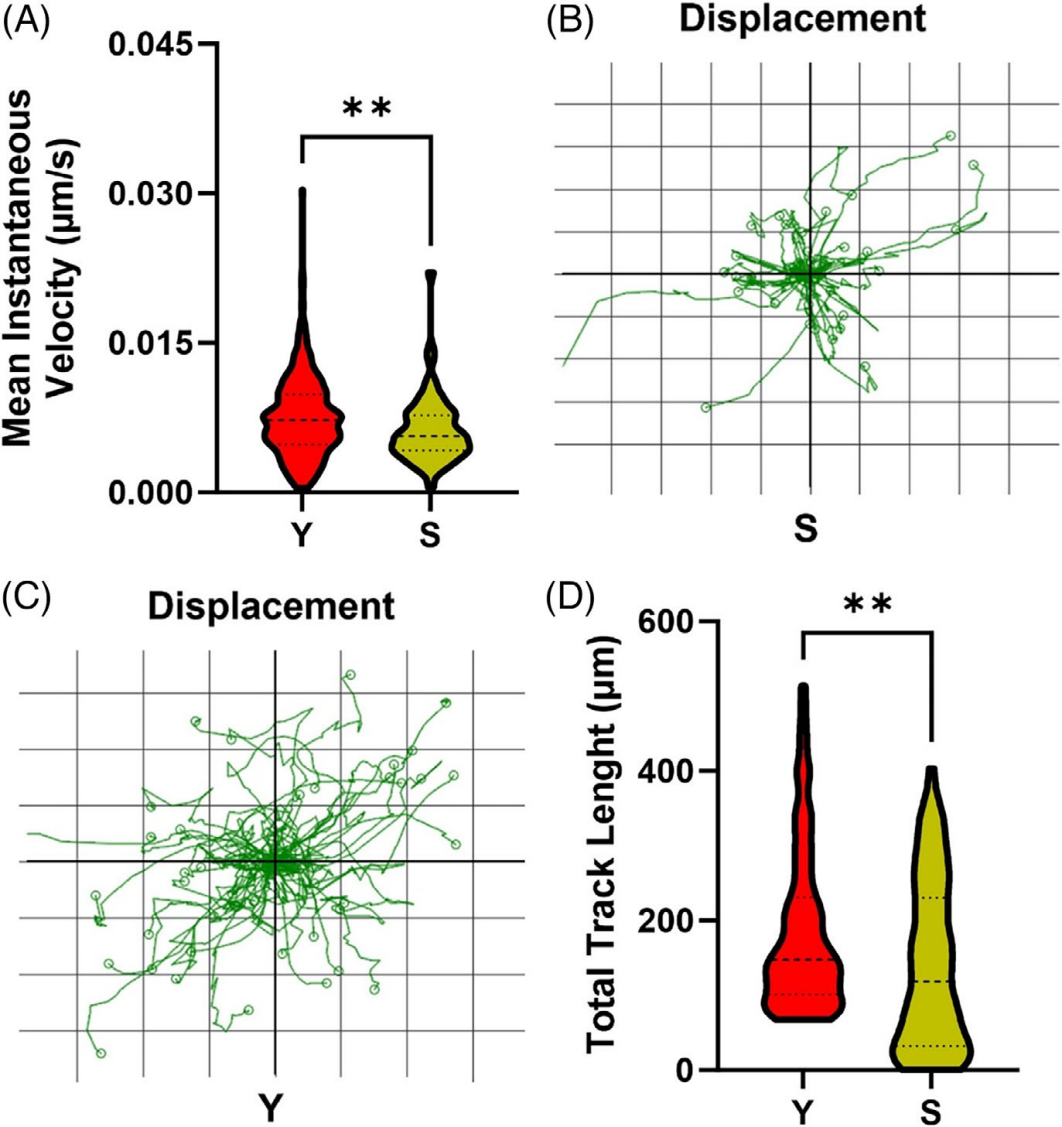
Evaluation of instantaneous velocity (A), displacement of (B) S and (C) Y, total track length (D) of hMSCs. The dashed lines within the violin distribution indicate the median value of the distributions while the dotted lines represent the quartiles. Significative differences between S and Y cells are indicated as **p < 0.05.

Videos of Y and S cells are provided in Supplementary Information S3, and in Figure 3, the analysis of cell motility is shown. In particular, we evaluated the median cell speed (as instantaneous velocity) (Figure 3A) and cell displacement, as depicted in Figure 3B for S cells and in Figure 3C for Y cells. In our samples, senescence significantly reduced cell motility, as indicated by a decrease in the average instantaneous velocity from 0.006 µm/s in Y cells to 0.004 µm/s in S cells and a more restricted displacement in the latter, as confirmed by the total track length (in Figure 3D).

## 4 DISCUSSION

The current study highlights the morphological and functional alterations associated with hMSC senescence by Quantitative Phase Imaging (QPI) using the Livecyte microscope.

Using a noninvasive, label‐free approach, this work provides valuable insights into the cellular changes that occur in MSC senescence, with potential implications for regenerative medicine.

The findings underscore significant differences in the morphology of young and senescent hMSCs. Notably, senescent cells exhibited a marked increase in area, being over three times larger than the young counterparts. This observation aligns with the well‐documented phenomenon of cellular hypertrophy in senescence, attributed to increased cytoplasmic and protein content, as well as alterations in the cytoskeleton.^15^, ^16^, ^29^ Additionally, changes in dry mass and volume were observed, with senescent hMSCs displaying a 3.4‐fold increase in dry mass compared to young cells. Interestingly, although these cells became larger, they were thinner and exhibited reduced sphericity, indicating that the remodelling processes occurring during senescence are rather complex.

The subtleties of single‐cell heterogeneity and the complex behaviours of cells can be captured through quantitative and temporal analysis approaches, as reported by Wiggins,^30^ providing cell phenotyping. These morphological changes observed in this study provide a basis for identifying senescent cells in culture and could serve as potential biomarkers for quality control in hMSCs‐based applications.

Reduced motility is another hallmark of senescent hMSCs, as evidenced by decreased instantaneous velocity, track speed and migratory speed in senescent compared to young cells. The decline in motility is impactful, given the critical role of MSC migration in bone health. Impaired motility may hinder the ability of hMSCs to home to injury sites or integrate effectively into target tissues, potentially contributing to the pathogenesis of bone diseases.

The observed morphological and functional changes in senescent hMSCs have relevant implications for their therapeutic use. Senescent cells are known to exhibit reduced proliferation, altered secretory profiles, and impaired differentiation potential,^31^ which can compromise the efficacy of MSC‐based therapies. Furthermore, the accumulation of senescent cells during long‐term culture could contribute to pro‐inflammatory and deleterious effects if transplanted into patients. Therefore, the ability to non‐invasively identify and potentially eliminate senescent cells is of paramount importance. The application of QPI, as demonstrated in this study, represents a promising tool for monitoring cellular health and optimising MSC cultures.

While this study provides valuable insights, some limitations should be addressed in future research. The observation period of 20 h might not capture long‐term dynamic changes in cellular behaviour. Extending the observation period could provide a more comprehensive understanding of senescence‐associated phenotype. Furthermore, the study primarily focuses on morphological metrics. Integrating functional assays, such as analyses of differentiation potential or secretory profiles, would provide a more holistic understanding of how senescence impacts MSC functionality. Moreover, the inclusion of additional techniques, such as transcriptomic or proteomic profiling, could elucidate the molecular mechanisms underlying the observed morphological changes.

## 5 CONCLUSIONS

This study demonstrates the utility of QPI as a powerful, noninvasive method to characterise senescence‐associated morphological changes in hMSCs. The identification of specific biomarkers, such as increased area and dry mass associated with reduced motility, provides a valuable framework to improve the quality and efficacy of MSC‐based therapies. Future research should focus on extending these findings to develop robust protocols for selective elimination of senescent cells and optimisation of MSC cultures for therapeutic applications.

## AUTHOR CONTRIBUTIONS

All authors contributed equally to this work. Specifically, Anconelli L., Farruggia G., Iotti S. and Maier J.A. conceptualised and designed the study; Anconelli L., Borsetti F., Rossi F. and Zafferri I. performed the experiments; Iotti S., Maier A.J., Rossi F., and Borsetti F. analysed the data; and Farruggia G., Anconelli L. and Maier A.J. wrote the manuscript. All authors reviewed and approved the final manuscript.

## Supporting information



Supporting Information 1

Supporting Information 2

Supporting Information 3

Supporting Information 4
