# Supplementary material for: Ptychographic analysis of human bone marrow‐derived mesenchymal stem cell morphology: The impact of cell senescence
Source: J Microsc. 2025 Jul 11;300(2):227–33. doi: 10.1111/jmi.70003 (PMC12523980; doi:10.1111/jmi.70003)
Supplement: Supplementary file 1 — Supporting Information 1 [file JMI-300-227-s003.docx]

ABSTRACT DIVULGATIVO

Human mesenchymal stem cells derived from bone marrow (hMSCs) are widely used in regenerative medicine due to their ability to repair tissues and promote healing. However, as these cells age, they lose some of their essential functions. In this study, we used an innovative technique called quantitative phase imaging (QPI) to observe and analyze how MSCs change during the aging process. Using the specialized Livecyte microscope, we non-invasively monitored changes in the size, shape, and movement of the cells. We found that aged cells are significantly larger, but they are thinner, less spherical, and less mobile compared to younger cells. These changes could limit their ability to migrate to damaged tissues and perform their repair functions. By providing detailed insights without the need for chemical treatments or dyes, this advanced imaging method offers a valuable tool for ensuring the quality and readiness of cells for medical applications.

Supplementary Information

**Area**, calculated for each of the segmented cells. It is the cumulative number of pixels included within a feature’s segmentation boundary, multiplied by the pixel size of the image.

**Length Width Ratio (LWR)**, calculated by dividing the length by the width of the smallest rectangular region in which the cells reside.

**Dry mass**, which is a measure of the total non-water cellular matter of a feature. It is directly related to the volume, however it attempts to mathematically remove all water content of a cells. Dry mass is calculated by the equation

$$Measured Phase Value x \lambda/(2*\pi*\left( refractive index Increment \right))$$

Where λ is the wavelength of the laser (651 nm), and the refractive index increment is set to a value of 0.2. These estimates are referenced from values in the literature [22].

To calculate the Dry mass of a feature, it has been summed up the dry mass of each pixel inside the feature.

**Volume,** calculated by summing up the optical volume of each individual pixel, calculated as area of the pixel multiplied by the optical thickness. Optical thickness is calculated by the equation:

$$Measured Phase Value x \lambda/(2*\pi*\left( cell refractive index-media refractive Index \right))$$

*Wher*e λ is the wavelength of the laser (651 nm), the cell refractive index is estimated as 1.38 and the media refractive index is estimated as 1.33. These estimates are referenced from values in the literature [22].

**Thickness** is a measure of the optical thickness of each cell, calculated by taking the Volume and dividing by the area of the segmented feature.

**Sphericity** is a measure of how close to a sphere a feature is. It is calculated by taking the volume of a feature, calculating the surface area of a sphere with that volume and dividing by the actual surface area of the Feature:

*Sph = (π^1/3^_*_6V^2/3^)/Surface area*

**Instantaneous Velocity,** calculated as the change of a position of a cell from frame (n-1) to frame (n) divided by time at frame(n) -time at frame (n.1).
