# Supplementary figures and images for: Ptychographic analysis of human bone marrow‐derived mesenchymal stem cell morphology: The impact of cell senescence
Source: J Microsc. 2025 Jul 11;300(2):227–33. doi: 10.1111/jmi.70003 (PMC12523980; doi:10.1111/jmi.70003)

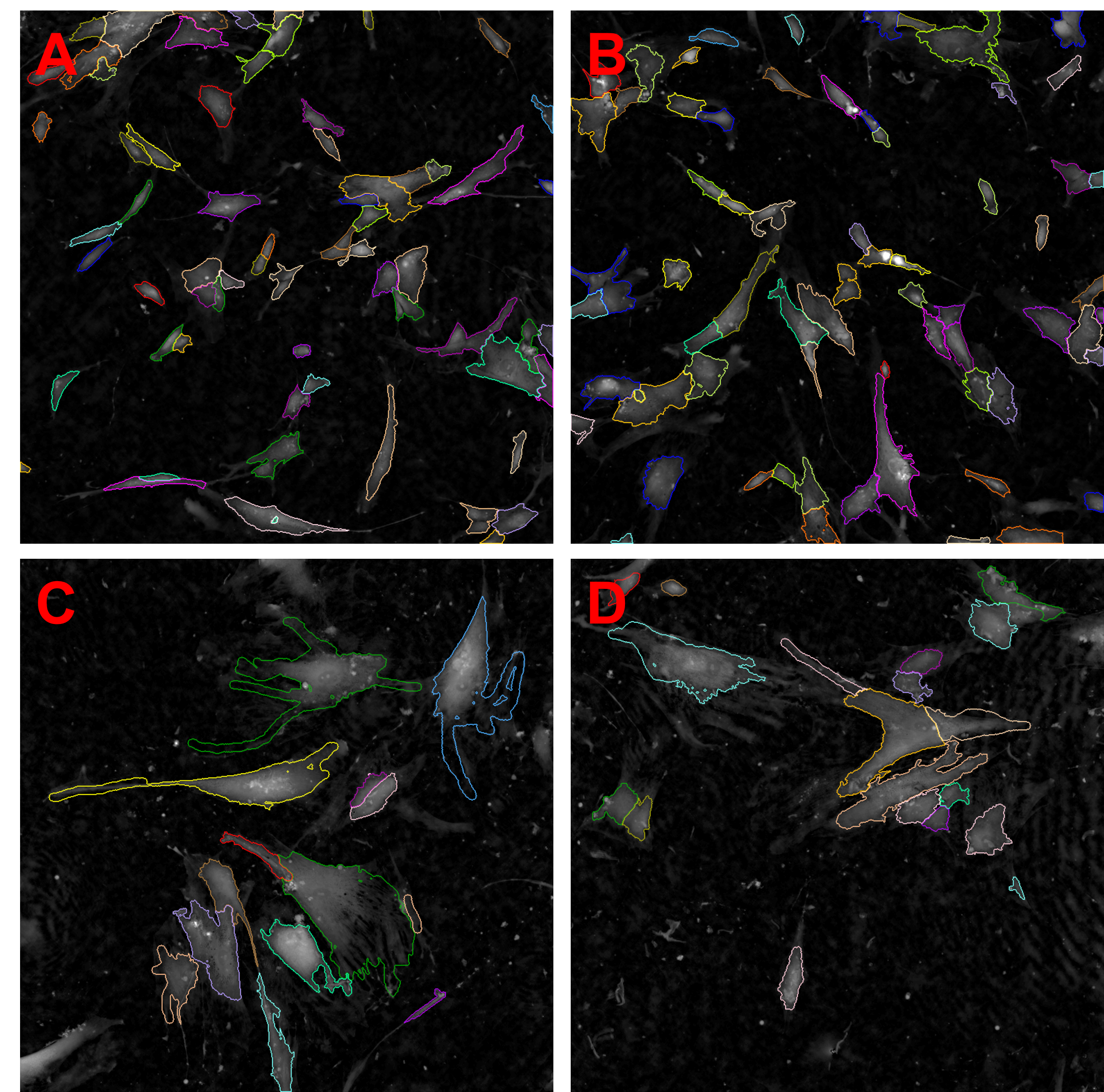

Supplement: Supplementary file 2 — Supporting Information 2 [file JMI-300-227-s004.tif]

## Slide 1
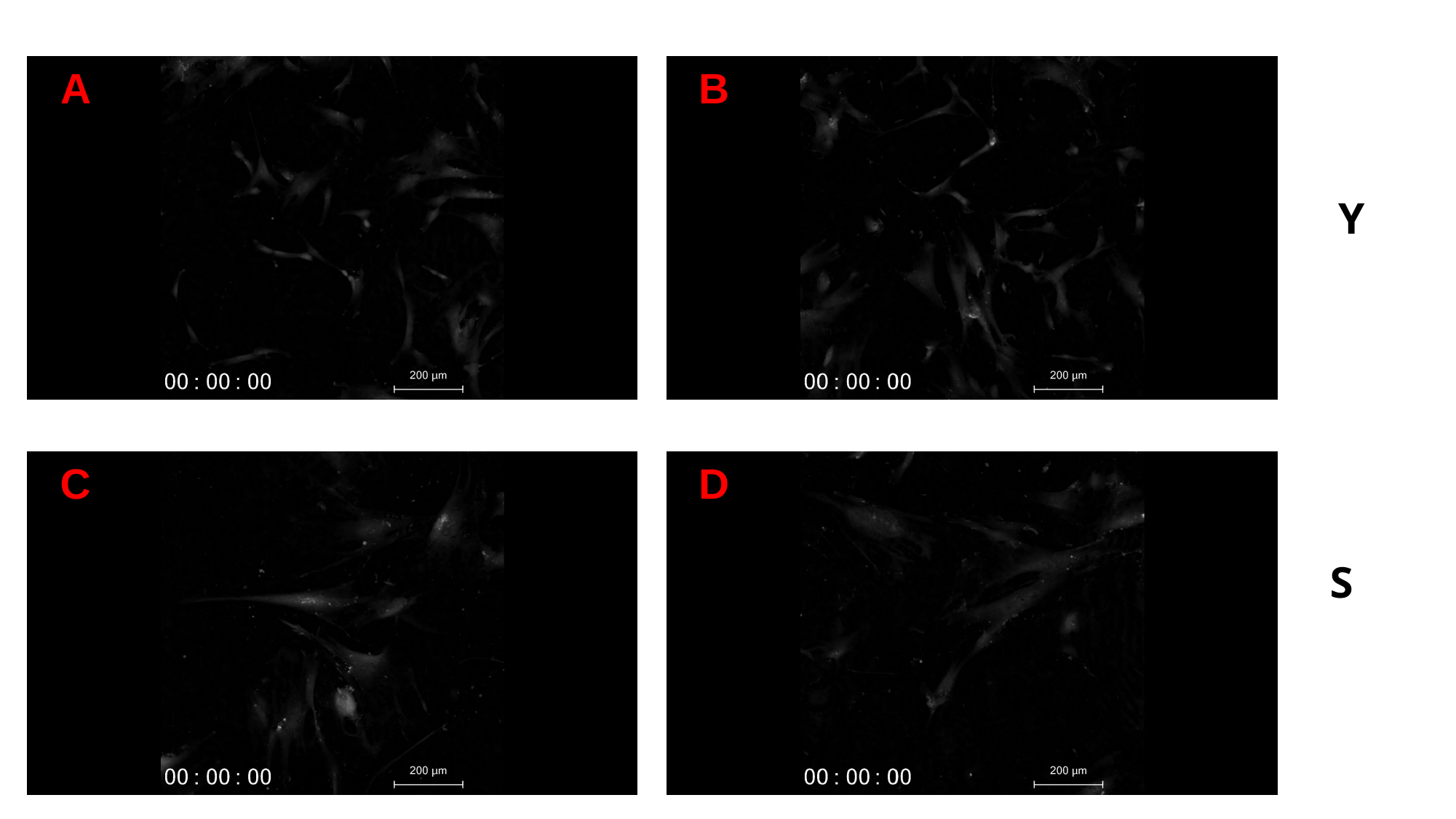

A
B
Y
C
D
S

Supplement: Supplementary file 4 — Supporting Information 4 [file JMI-300-227-s002.pptx]
